# Quantitative Analysis of Myocardial Work by Non-invasive Left Ventricular Pressure-Strain Loop in Patients With Type 2 Diabetes Mellitus

**DOI:** 10.3389/fcvm.2021.733339

**Published:** 2021-10-01

**Authors:** Danqing Huang, Cunying Cui, Qiang Zheng, Yanan Li, Yuanyuan Liu, Yanbin Hu, Ying Wang, Ruijie Liu, Lin Liu

**Affiliations:** ^1^Department of Ultrasound, Fuwai Central China Cardiovascular Hospital, Central China Fuwai Hospital of Zhengzhou University, Henan Provincial People's Hospital, People's Hospital of Zhengzhou University, Zhengzhou, China; ^2^School of Computer and Control Engineering, Yantai University, Yantai, China

**Keywords:** type 2 diabetes mellitus, echocardiography, myocardial work, pressure-strain loop, left ventricle function

## Abstract

**Background:** Type 2 diabetes mellitus (T2DM) is a common risk factor for cardiovascular diseases. The aims of this study were to evaluate the changes in the left ventricular myocardial work in T2DM patients using the left ventricular pressure-strain loop (PSL) technique, and to explore the risk factors for the left ventricular myocardial work impairment.

**Methods:** Fifty patients with T2DM and 50 normal controls (NCs) were included in the study. In addition to conventional echocardiography and two-dimensional speckle tracking echocardiography, the left ventricular myocardial work parameters were measured using PSL technology.

**Results:** The absolute value for global longitudinal strain (GLS), global work index (GWI) and, global constructive work (GCW) were significantly decreased in the T2DM group (*P* < 0.05), while the left ventricular ejection fraction (LVEF) was not significantly different between the T2DM and NC groups. Multivariable linear regression analysis showed that hemoglobin A1c (HbA1c) was independently related to GWI (β = −0.452, *P* < 0.05), while HbA1c and the diabetes duration were independently related to GCW (β = −0.393, *P* < 0.05 and β = −0.298, *P* < 0.05, respectively).

**Conclusions:** Changes in the left ventricular myocardial systolic function in T2DM patients were identified using PSL technology. HbA1c was shown to be an independent risk factor affecting GWI, while HbA1c and diabetes duration were demonstrated to be independent risk factors affecting GCW.

## Introduction

Diabetes is a common clinical metabolic disease. Type 2 diabetes mellitus (T2DM) accounts for more than 90% of diabetic patients (1). The incidence of cardiovascular disease is 2-3 times higher in T2DM patients compared to healthy individuals (2). Therefore, the evaluation of left ventricular systolic function in T2DM patients at an early stage is important for treatment and prognosis.

Left ventricular ejection fraction (LVEF) and two-dimensional speckle tracking technology are commonly used to evaluate the left ventricular systolic function, but these methods are load-dependent (3). Recently, a new non-invasive left ventricular pressure-strain loop (PSL) technology has been developed based on two-dimensional speckle tracking technology to assess the changes in left ventricular myocardial function. PSL technology combines the ventricular deformation and pressure, and the influence of cardiac afterload on traditional myocardial strain measurement is considered (4–6). Hubert et al. invasively measured the left ventricular pressure in patients implanted with a bi-ventricular pace-maker, and blood pressure was measured by brachial artery cuff-pressure for estimating the left ventricular pressure. They found that the maximum systolic value was different between measured and estimated pressures, but the estimated and measured PSL and global myocardial work indices were strongly correlated, with an R^2^ > 0.88. The major reason is that the temporal integration induces a smoothing of the difference between measured and estimated works, so the deducted estimation of left ventricular work is accurate (7).

A previous study showed that the level of hemoglobin A1c (HbA1c) was independently associated with decreased left ventricular strain in T2DM patients with preserved LVEF (8). However, the relationship between HbA1c and left ventricular PSL parameters in T2DM patients with normal LVEF is unclear. We hypothesized that HbA1c is independently associated with left ventricular PSL parameters in T2DM patients with normal LVEF.

The aims of the present study were to evaluate the changes in the left ventricular myocardial work in T2DM patients using the left ventricular PSL technique, and to explore the clinical factors impairing the left ventricular myocardial work.

## Methods

### Study Population

**Figure 1:**
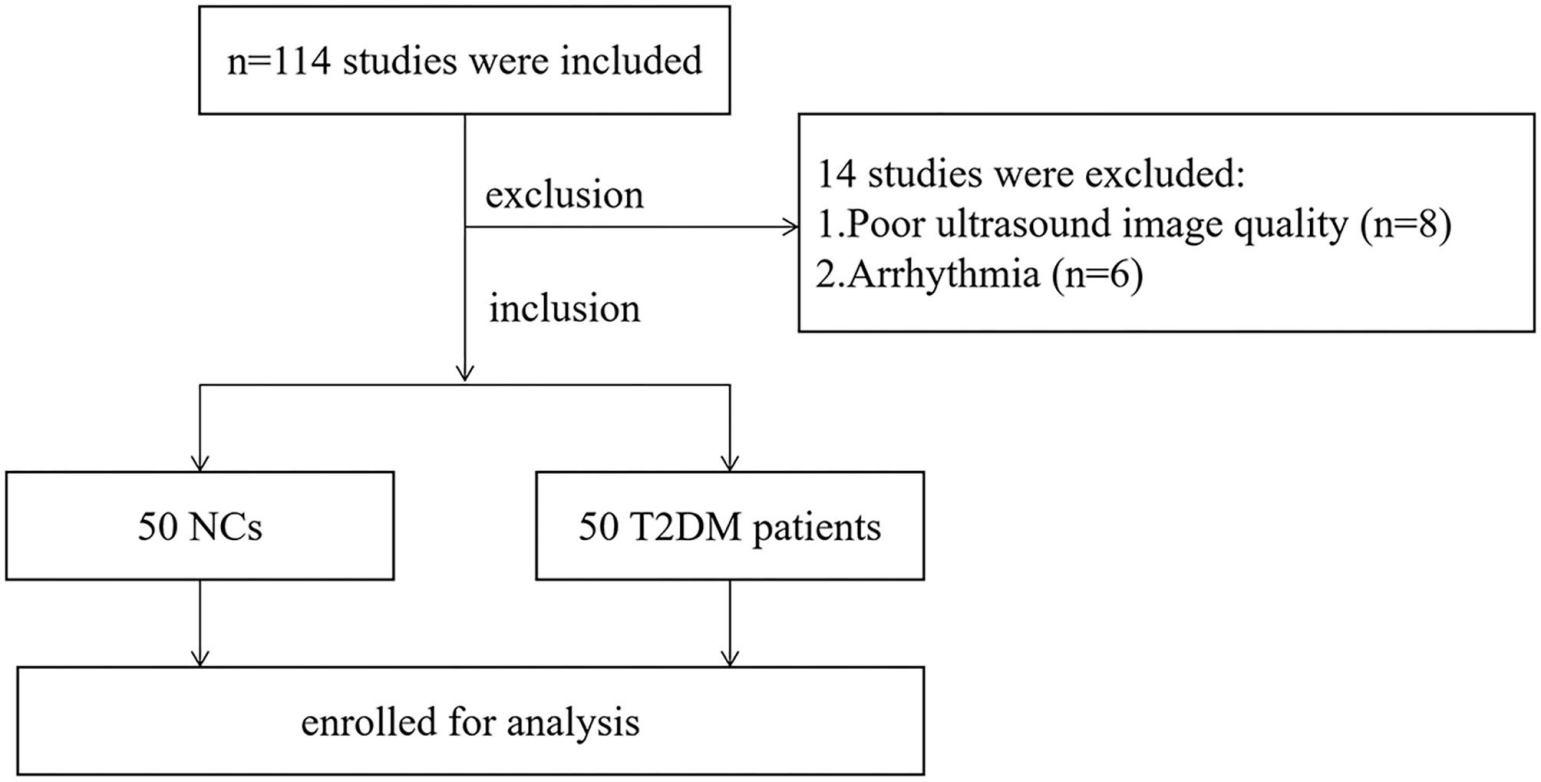
Flow chart of participants inclusion in the T2DM and NC groups. T2DM, type 2 diabetes mellitus; NC, normal control.

A total of 100 participants were consecutively enrolled in Fuwai Central China Cardiovascular Hospital between May and December of 2020, of which 50 were in the T2DM group (26/24, male/female) and 50 age- and gender-matched healthy individuals were in the normal control (NC) group (29/21, male/female) (Figure 1). The inclusion criteria for all T2DM patients were set according to the 2020 guidelines of the American Diabetes Association (9). Participants with any one of the following conditions were excluded: LVEF < 50%, heart failure, arterial hypertension, valvular heart disease, arrhythmia, congenital heart disease, or poor ultrasound image quality. In view of relatively higher incidence of coronary heart disease in T2DM patients, the recent examination of coronary angiography or computerized tomography were performed in all participants. Those who suffered from obvious atherosclerotic stenosis should also be excluded. The study was approved by the ethics committee of Fuwai Central China Cardiovascular Hospital and informed consents was obtained before participation.

### Laboratory Analyses

Total cholesterol (TC), triglyceride (TG), high-density lipoprotein cholesterol (HDL-C), low-density lipoprotein cholesterol (LDL-C), and HbA1c levels were measured <2 weeks before the echocardiographic evaluation using standard laboratory techniques. Fasting plasma glucose (FPG) and 2-h postprandial plasma glucose (2h-PPG) levels were measured in T2DM patients.

### Echocardiographic Examination

Transthoracic echocardiography was performed using a Vivid E95 system (GE Vingmed Ultrasound AS, Horten, Norway) equipped with an M5Sc-D probe (1.4-4.6 MHz). All study participants were scanned in the left lateral position with continuous electrocardiogram monitoring. Left atrial diameter (LAD), left ventricular end diastolic diameter (LVDd), left ventricular end diastolic volume (LVEDV), left ventricular end systolic diameter (LVDs), left ventricular end systolic volume (LVESV), and LVEF were measured in the parasternal long-axis view of the left ventricle. Doppler spectrum images of the aortic and mitral valves were collected from apical five- and four-chamber views. Two-dimensional images consisting of three cardiac cycles from the apical four-, three-, and two-chamber views were acquired at frame rates between 57 and 68 frames/s (mean, 65 ± 6 frames/s). All images were stored on a hard disk for offline analysis.

### Left Ventricular Strain and Myocardial Work Quantification

**Figure 2:**
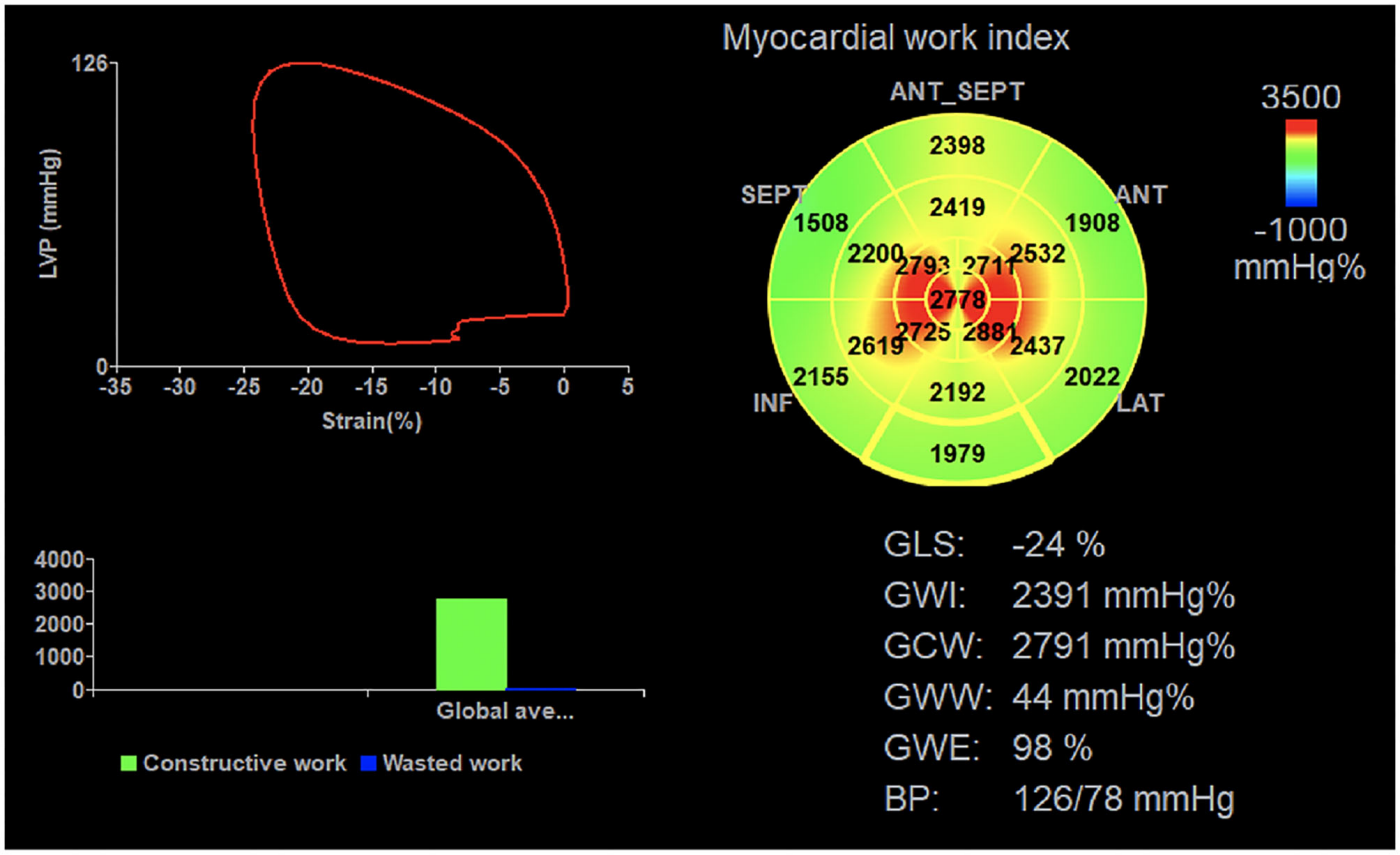
Left ventricular myocardial work parameters were measured using the non-invasive PSL technique. Cuff contraction pressure is represented on the ordinate and longitudinal strain on the abscissa. PSL, pressure-strain loop; GLS, global longitudinal strain; GWI, global work index; GCW, global constructive work; GWW, global wasted work; GWE, global work efficiency, BP blood pressure.

Echopac version 203 software (GE vingmed ultra sound, Horten, Norway) was used for image analysis. Three index points were used to define the mitral annulus and left ventricular apex at the end-systolic frame in each apical view. Automated tracking of myocardial motion was performed with the region of interest adjusted by correcting the endocardial border or width if necessary. The software calculated global longitudinal strain (GLS) from the weighted average of the peak systolic longitudinal strain of the 17 segments. Peak systolic left ventricular pressure was assumed to be equal to the peak arterial pressure, which was recorded from the brachial cuff blood pressure measured immediately before the echocardiographic recordings. A non-invasive left ventricular pressure curve was constructed using the strain and blood pressure data, and a normalized reference curve adjusted according to the duration of isovolumic and ejection phases defined by the timing of aortic and mitral valve opening and closing events on Doppler spectrum images (10). Left ventricular myocardial work parameters was subsequently computed by the differentiation of the strain values over time multiplying the instantaneous LV pressure (Figure 2). The myocardial work parameters are as follows:

1. Global work index (GWI): total work within the area of the left ventricular PSL calculated from mitral valve closure to mitral valve opening.
2. Global constructive work (GCW): work performed by during left ventricular shortening in systole and lengthening during the isovolumic relaxation phase.
3. Global wasted work (GWW): the negative work performed during left ventricular lengthening in systole and shortening in isolvolumic relaxation phase.
4. Global work efficiency (GWE): the percentage of constructive work divided by the sum of constructive and wasted work.

### Statistical Analysis

The statistical analyses were performed using SPSS 26.0 software (IBM, Armonk, NY, USA). *P*-value <0.05 was considered statistically significant. Continuous variables were expressed as mean ± standard deviation for normally distributed data, or as median (interquartile range) for non-normally distributed data. Categorical variables were expressed as frequencies and percentages.

The statistical analysis was performed using the Student's *t*-test, Mann-Whitney *U*-test, and χ^2^ test as appropriate to compare the T2DM and NC groups. Pearson's correlation coefficient was used for determining the correlation between GLS and myocardial work parameters. The clinical factors with *P*-value <0.05 in univariable linear regression outcomes were incorporated into the multivariable linear regression analysis models by means of stepwise selection to detect the independent predictors of abnormal myocardial function in T2DM patients. There was no multicollinearity between variables in these models. Ten subjects were randomly selected and re-measured the global myocardial work parameters by two observers. Intra-observer and inter-observer variability was assessed in 20 randomly selected subjects using the Bland-Altman analyses.

## Results

### Study Population Characteristics

Body mass index (BMI), body surface area (BSA), HbA1c, TG, and LDL-C in the T2DM group were significantly increased compared to the NC group (*P* < 0.05) (Table 1). There were no significant differences in gender, age, heart rate, systolic blood pressure (SBP), diastolic blood pressure (DBP), TC, HDL-C, and smoking history between the two groups (*P* > 0.05).

**Table 1:** Statistical comparisons of demographic characteristics and clinical parameters between NC and T2DM groups.

**Parameters**	**NC group *n* = 50**	**T2DM group *n* = 50**		
			***χ*** ^**2**^	***P-*** **value**
Male gender, *n* (%)	29 (58%)	26 (52%)	0.364	0.546
Smoking, *n* (%)	12 (24%)	14 (28%)	0.208	0.648
			***t***	***P-*** **value**
Age (years)	46.88 ± 10.60	50.20 ± 9.73	−1.632	0.106
BMI (kg/m^2^)	24.32 ± 3.67	26.09 ± 3.80	−2.370	0.020
BSA (m^2^)	1.72 ± 0.16	1.83 ± 0.24	−2.449	0.016
SBP (mm Hg)	118.84 ± 3.94	120.68 ± 6.00	−1.813	0.073
DBP (mm Hg)	77.70 ± 6.31	79.24 ± 7.40	−1.120	0.265
pulse pressure (mm Hg)	41.14 ± 7.13	41.44 ± 8.85	−0.187	0.852
Heart rate (bpm)	67.82 ± 8.37	71.02 ± 9.41	−1.797	0.075
TC (mmol/L)	4.33 ± 0.61	4.50 ± 1.01	−0.957	0.341
TG (mmol/L)	1.65 ± 0.64	2.17 ± 1.04	−2.296	0.004
LDL-C (mmol/L)	2.48 ± 0.70	2.79 ± 0.66	−2.196	0.030
HbA1c (%)	5.24 ± 0.36	8.06 ± 1.37	−10.350	<0.001
			***Z***	***P-*** **value**
HDL-C (mmol/L)	1.01 (0.30)	1.03 (0.23)	−0.259	0.796
FPG (mmol/L)	–	7.88 ± 1.49	–	–
2h-PPG (mmol/L)	–	11.60 ± 2.32	–	–
Diabetes duration (years)	–	2.50 (9.37)	–	–
Oral antihyperglycemic agent, *n* (%)	–	23 (46%)	–	–
Insulin, *n* (%)	–	3 (6%)	–	–
Oral antihyperglycemic agent + insulin, *n* (%)	–	14 (28%)	–	–
Complications, *n* (%)	–	11 (22%)	–	–
Cardiovascular medications, *n* (%)	–	13 (26%)	–	–

*T2DM, type 2 diabetes mellitus; NC, normal control; BMI, body mass index;BSA, body surface area; SBP, systolic blood pressure; DBP, diastolic blood pressure; HbA1c, hemoglobin A1c; FPG, fasting plasma glucose; 2h-PPG, 2-h postprandial plasma glucose; TC, total cholesterol; TG, triglyceride; HDL-C, high-density lipoprotein cholesterol; LDL-C, low-density lipoprotein cholesterol*.

### Comparison of Conventional Echocardiographic Parameters and GLS

The absolute value of GLS in the T2DM group was significant lower compared to the NC group (*P* < 0.05). No significant difference was observed in LAD, LVDd, LVEDV, LVDs, LVESV, and LVEF between the T2DM and NC groups (*P* > 0.05) (Table 2).

**Table 2:** Statistical comparisons of conventional echocardiographic parameters and GLS between NC and T2DM groups.

**Parameters**	**NC group *n* = 50**	**T2DM group *n* = 50**		
			***t***	***P-*** **value**
LAD (mm)	33.38 ± 3.46	34.62 ± 3.45	−1.795	0.076
LVDd (mm)	45.76 ± 2.62	45.54 ± 2.11	0.463	0.645
LVEDV (ml)	98.00 ± 12.58	96.80 ± 10.68	0.514	0.608
			***Z***	***P-*** **value**
LVDs (mm)	30.00 (2.00)	30.00 (2.00)	−0.267	0.790
LVESV (ml)	35.50 (5.00)	35.00 (6.00)	−0.253	0.801
LVEF (%)	63.50 (3.25)	62.00 (4.00)	1.552	0.121
GLS (%)	−19.50 (3.00)	−17.00 (3.00)	−4.509	<0.001

*T2DM, type 2 diabetes mellitus; NC, normal control; LAD, left atrial diameter; LVDd, left ventricular end diastolic diameter; LVEDV, left ventricular end diastolic volume; LVDs, left ventricular end systolic diameter; LVESV, left ventricular end systolic volume; LVEF, left ventricular ejection fraction; GLS, global longitudinal strain*.

### Comparison of Myocardial Work

**Figure 3:**
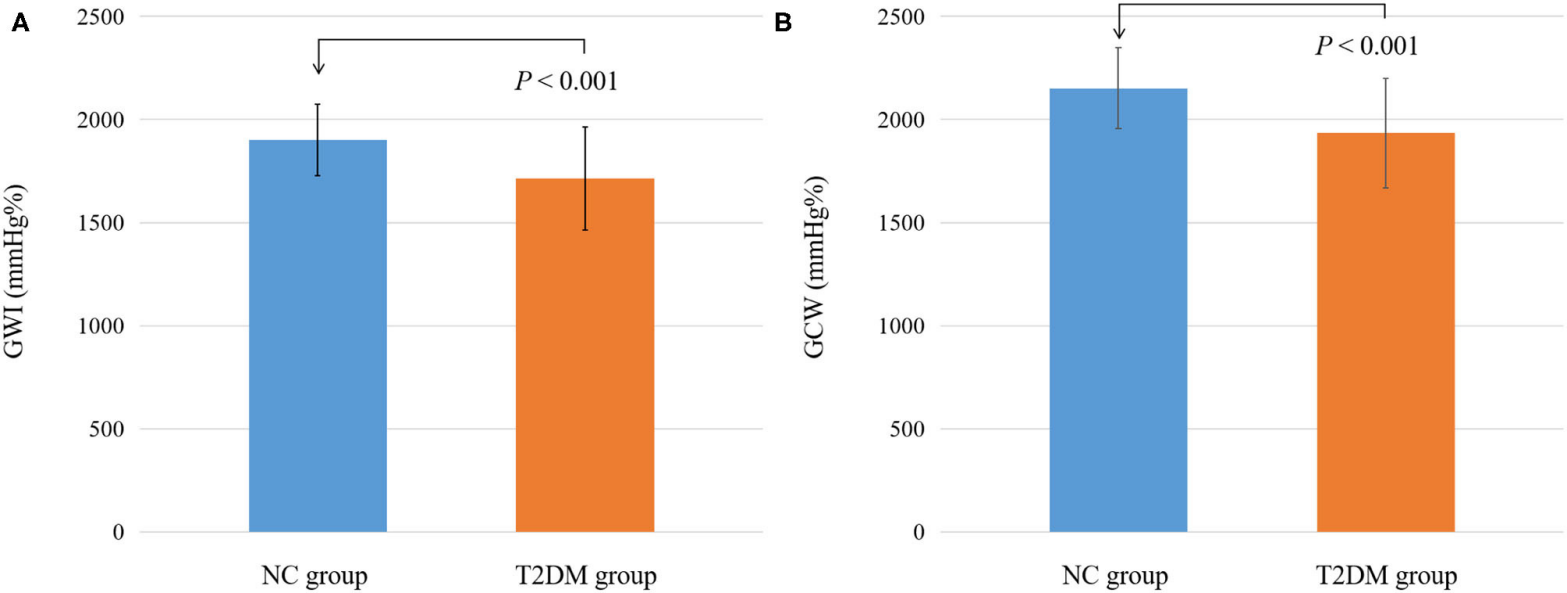
Differential changes of myocardial work parameters between NC and T2DM groups. **(A)** Differential changes of GWI between NC and T2DM groups. **(B)** Differential changes of GCW between NC and T2DM groups.T2DM, type 2 diabetes mellitus; NC, normal control; GWI, global work index; GCW, global constructive work.

The GWI and GCW in the T2DM group were significantly decreased compared to the NC group (*P* < 0.05) (Table 3; Figure 3). However, there were no significant differences in GWW and GWE between the two groups (*P* > 0.05).

**Table 3:** Statistical comparisons of global myocardial work parameters between NC and T2DM groups.

**Parameters**	**NC group *n* = 50**	**T2DM group *n* = 50**		
			***t***	***P-*** **value**
GWI (mm Hg%)	1899.84 ± 173.47	1712.80 ± 249.44	4.353	<0.001
GCW (mm Hg%)	2151.08 ± 196.17	1934.58 ± 266.64	4.625	<0.001
			***Z***	***P-*** **value**
GWW (mm Hg%)	34.50 (26.50)	45.00 (38.50)	−1.421	0.155
GWE (%)	98.00 (1.00)	97.00 (2.00)	−1.848	0.065

*T2DM, type 2 diabetes mellitus; NC, normal control; GWI, global work index; GCW, global constructive work; GWW, global wasted work; GWE, global work efficiency*.

### Correlation Between GLS and Myocardial Work Parameters

**Figure 4:**
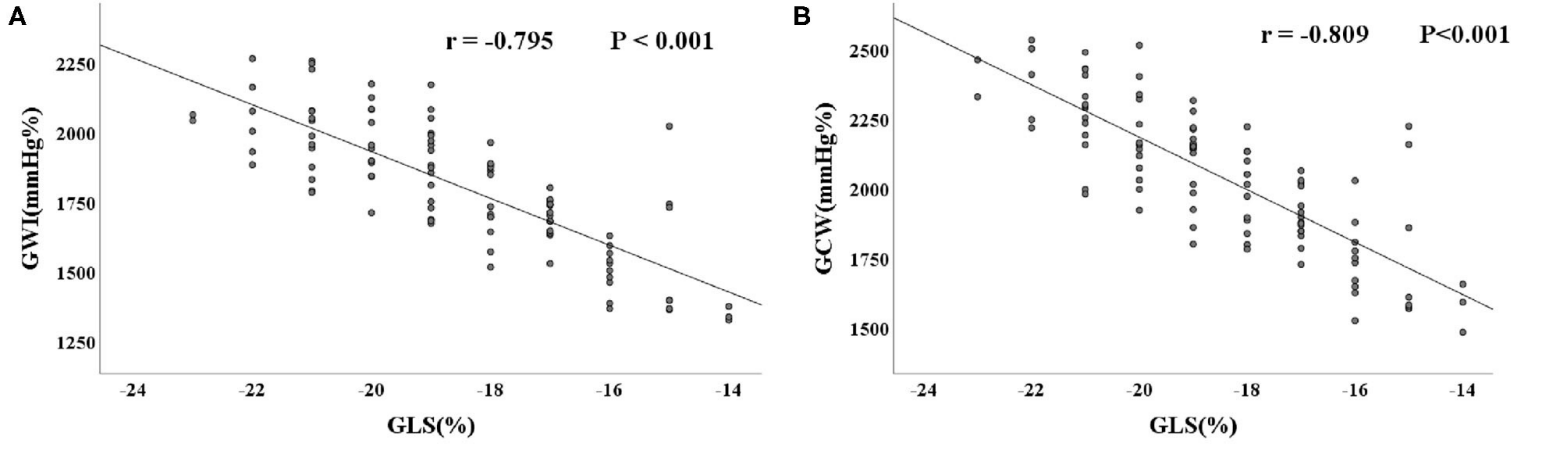
Correlation between myocardial work parameters and GLS. **(A)** Correlation between GWI and GSL. **(B)** Correlation between GCW and GSL. PSL, pressure-strain loop; GLS, global longitudinal strain; GWI, global work index; GCW, global constructive work.

GLS showed a good correlation with GWI and GCW (*r* = −0.795, *P* < 0.001 and *r* = −0.809, *P* < 0.001, respectively) (Figure 4).

### Risk Factor Analysis for Left Ventricular Myocardial Work Impairment in T2DM Patients

BSA, HbA1c, FPG, and diabetes duration were incorporated into the multivariate linear regression model of GWI and GCW by means of stepwise selection based on the univariate linear regression analysis results. HbA1c was independently associated with GWI, while HbA1c and the diabetes duration were independently associated with GCW (*P* < 0.05) (Tables 4, 5).

**Table 4:** Univariable and multivariable linear regression analysis of GWI in T2DM patients.

	**Univariable analysis**		**Multivariable analysis**	
	**β**	***P–*value**	**β**	***P–*value**
Age (years)	−0.065	0.654	–	–
BSA (m^2^)	−0.340	0.016	–	–
Heart rate (bpm)	−0.191	0.183	–	–
SBP (mm Hg)	0.178	0.217	–	–
DBP (mm Hg)	−0.130	0.367	–	–
Pulse pressure (mm Hg)	0.229	0.109	–	–
TC (mmol/L)	−0.209	0.149	–	–
TG (mmol/L)	0.060	0.677	–	–
HDL–C (mmol/L)	0.123	0.397	–	–
LDL–C (mmol/L)	−0.205	0.152	–	–
HbA1c (%)	−0.452	0.001	−0.452	−0.001
FPG (mmol/L)	−0.304	0.032	–	–
2h–PPG (mmol/L)	−0.124	0.391	–	–
Diabetes duration (years)	−0.357	0.011	–	–
R^2^				0.204
Adjusted R^2^				0.187

*T2DM, type 2 diabetes mellitus; BSA, body surface area; SBP, systolic blood pressure; DBP, diastolic blood pressure; HbA1c, hemoglobin A1c; FPG, fasting plasma glucose; 2h–PPG, 2–h postprandial plasma glucose; TG, triglyceride; TC, total cholesterol; HDL–C, high–density lipoprotein cholesterol; LDL–C, low–density lipoprotein cholesterol; GWI, global work index; R^2^, coefficient of determination; β, standardized regression coefficients*.

**Table 5:** Univariable and multivariable linear regression analysis of GCW in T2DM patients.

	**Univariable analysis**		**Multivariable analysis**	
	**β**	***P-*value**	**β**	***P-*value**
Age (years)	−0.192	0.183	–	–
BSA (m^2^)	−0.355	0.011	–	–
Heart rate (bpm)	−0.135	0.349	–	–
SBP (mm Hg)	0.210	0.143	-	–
DBP (mm Hg)	−0.080	0.579	–	–
Pulse pressure (mm Hg)	0.209	0.144	–	–
TC (mmol/L)	−0.130	0.369	–	–
TG (mmol/L)	0.123	0.395	–	–
HDL-C (mmol/L)	0.125	0.385	–	–
LDL-C (mmol/L)	−0.206	0.151	–	–
HbA1c (%)	−0.517	<0.001	−0.393	0.004
FPG (mmol/L)	−0.347	0.013	–	–
2h-PPG (mmol/L)	−0.153	0.289	–	–
Diabetes duration (years)	−0.461	0.001	−0.298	0.027
R^2^				0.340
Adjusted R^2^				0.312

*T2DM, type 2 diabetes mellitus; BSA, body surface area; SBP, systolic blood pressure; DBP, diastolic blood pressure; HbA1c, hemoglobin A1c; FPG, fasting plasma glucose; 2h-PPG, 2-h postprandial plasma glucose; TG, triglyceride; TC, total cholesterol; HDL-C, high-density lipoprotein cholesterol; LDL-C, low-density lipoprotein cholesterol; GCW, global constructive work; R^2^, coefficient of determination; β, standardized regression coefficients*.

### Reproducibility Test

Intra-observer and inter-observer variability for global myocardial work parameters are summarized in Table 6. Bland-Altman analyses showed good repeatability and reproducibility in global myocardial work parameters.

**Table 6:** Repeatability and reproducibility of myocardial work parameters.

**Parameters**	**Mean ± SD**	**Mean ± SD**	**Bias**	**95%LOA**
**Intraobserver**				
GWI (mm Hg%)	1,784.70 ± 248.05	1,797.40 ± 239.75	20.70	−50.57–91.97
GCW (mm Hg%)	1,965.95 ± 262.88	2,009.05 ± 262.523	43.10	−128.58–214.78
GWW (mm Hg%)	36.65 ± 15.28	39.90 ± 16.37	3.25	−4.96–11.46
GWE (%)	97.40 ± 0.88	97.45 ± 0.76	0.05	−0.95–1.05
**Interobserver**				
GWI (mm Hg%)	1,784.70 ± 248.05	1,783.65 ± 220.39	−8.35	−128.20–111.50
GCW (mm Hg%)	1,965.95 ± 262.88	1,991.60 ± 258.02	25.65	−110.66–161.96
GWW (mm Hg%)	36.65 ± 15.28	38.75 ± 12.78	2.30	−10.29–14.89
GWE (%)	97.40 ± 0.88	97.25 ± 0.79	−0.15	−1.11–0.81

*SD, standard deviation; LOA, limits of agreement; SD, standard deviation. GWI, global work index; GCW, global constructive work; GWW, global wasted work; GWE, global work efficiency*.

## Discussion

The main findings of this study showed that GWI and GCW were significantly different between the NC and T2DM groups. PSL technology was able to assess the impairment of left ventricular systolic function in T2DM patients. Multivariable linear regression analysis confirmed that HbA1c was independently related to GWI, while HbA1c and diabetes duration were independently related to GCW.

The HbA1c level in the T2DM group was higher than that in the NC group. HbA1c has been widely used as an indicator of diabetes control and it is correlated with FPG and 2h-PPG (11, 12). T2DM patients often suffer from insulin deficiency or insulin resistance, and the body fails to make full use of glucose to produce energy. Then, the lipid and protein metabolism is enhanced, resulting in weight loss. However, our results showed that the BMI in the T2DM group was higher than that in the NC group. This finding may be related to individual lifestyle and medication regimen (13). In addition, the levels of TG and LDL-C in the T2DM group were higher than those in the NC group. The free fatty acids produced by TG were able to further reduce insulin sensitivity, forming a vicious circle between TG levels and insulin resistance (14–16).

The absolute value of GLS in the T2DM group was lower compared to that in the NC group, while the LVEF was similar. Consistent with previous studies (17, 18), this result indicates that the left ventricular longitudinal systolic function is impaired in T2DM patients, and GLS is more sensitive than LVEF in reflecting the subtle change in myocardial function.

Several prior studies have shown that T2DM is closely related to vascular arteriosclerosis, which can elevate pulse pressure (19, 20). A widened pulse pressure may increase left ventricular afterload (21). Tadic M et al. found GWI was higher in hypertensive patients than in controls, and even higher in subjects with both hypertension and diabetes (22). Interestingly, our results showed GWI and GCW in T2DM patients were significantly reduced. This finding may be related to pulse pressure. We have excluded patients with hypertension in our study. Our results showed that there was no significant difference in pulse pressure between NC and T2DM groups. Therefore, the increase of the left ventricular afterload and the compensatory increase of the left ventricular pump function were insignificant. In our study, there was no significant difference in LVEF between T2DM patients and normal control group, but the absolute value of GLS, GWI, and GCW were significantly decreased in the T2DM group. Considering the influence of afterload on strain measurement results, PSL technology combined with ventricular strain and arterial pressure is more accurate in evaluating left ventricular systolic function than using GLS technology alone (23). Oberhoffer FS et al. showed that blood pressure and GLS were not significantly difference between Turner syndrome patients and healthy controls, but the GWI and GCW were significantly higher in Turner syndrome patients (24). Therefore, PSL technology is more sensitive and comprehensive than global strain and LVEF in evaluating early impairment of ventricular function. We also found that GLS have a good correlation with GWI and GCW. Reproducibility testing results for GWI, GCW, GWW, and GWE in the present study suggested a good repeatability.

HbA1c is an independent risk factor affecting GWI, while HbA1c and the diabetes duration are the independent risk factors affecting GCW, which may be related to the long-term hyperglycaemic environment. The potentially pathogenic conditions, such as endothelial dysfunction and oxidative stress, may impair the left ventricular systolic function (25–27). Previous studies have shown that diabetes duration is independently related to left ventricular diastolic dysfunction (28, 29). Another important finding in the present study was that the diabetes duration is independently related to GCW, further confirming that the diabetes course can also lead to left ventricular systolic dysfunction.

## Limitations

The limitations of this study are as follows. The present investigation was a cross-sectional research study performed at a single center with a relatively small sample size. PSL technology is based on two-dimensional speckle tracking imaging, which requires a high-quality ultrasound image. The follow-up data for prognostic effects in T2DM patients are lacking, so the relationship between PSL related parameters and left ventricular systolic dysfunction needs further longitudinal study.

## Conclusions

In conclusion, combined with the cardiac afterload, PSL technology can evaluate the changes in the left ventricular myocardial systolic function in T2DM patients with normal LVEF. HbA1c is an independent risk factor affecting GWI, while HbA1c and diabetes duration are the independent risk factors affecting GCW. Therefore, T2DM patients should be treated as soon as possible and the HbA1c level should be strictly controlled.

## Data Availability Statement

The raw data supporting the conclusions of this article will be made available by the authors, without undue reservation.

## Ethics Statement

The studies involving human participants were reviewed and approved by the Ethics Committee of Fuwai Central China Cardiovascular Hospital. The patients/participants provided their written informed consent to participate in this study.

## Author Contributions

DH and CC designed the study, analyzed the data, and wrote the manuscript. CC, YLi, YLiu, YH, YW, and RL assisted recruitment and manuscript revision. LL, QZ, and CC assisted in study design, data interpretation, and manuscript revision. All authors had read and approved the final manuscript.

## Funding

This study was supported by National Natural Science Foundation of China (82071950), National Natural Science Foundation of Henan for Excellent Young Scientists (202300410364), Medical Science and Technology Project of Henan Province (SB201901099), and Henan Provincial Medical Science and Technology Research Project (LHGJ20190805 and LHGJ20200084).

## Conflict of Interest

The authors declare that the research was conducted in the absence of any commercial or financial relationships that could be construed as a potential conflict of interest.

## Publisher's Note

All claims expressed in this article are solely those of the authors and do not necessarily represent those of their affiliated organizations, or those of the publisher, the editors and the reviewers. Any product that may be evaluated in this article, or claim that may be made by its manufacturer, is not guaranteed or endorsed by the publisher.
