# Supplementary material for: Quantitative Analysis of Myocardial Work by Non-invasive Left Ventricular Pressure-Strain Loop in Patients With Type 2 Diabetes Mellitus
Source: Front Cardiovasc Med. 2021 Oct 1;8:733339. doi: 10.3389/fcvm.2021.733339 (PMC8517392; doi:10.3389/fcvm.2021.733339)
Supplement: Supplementary file 1 [file Data_Sheet_1.docx]

**SUPPLEMENTAL TABLE 1 │**Statistical comparisons of global myocardial work parameters between non-complication and complication groups in T2DM

| **Parameters** | **non-complication group**  **n=39** | **complication group**  **n=11** |  |  |
| --- | --- | --- | --- | --- |
|  |  |  | ***t*** | ***P-*value** |
| GWI (mm Hg%) | 1719.03 ± 242.18 | 1690.73 ± 285.14 | -0.329 | 0.743 |
| GCW (mm Hg%) | 1941.08 ± 261.21 | 1911.55 ± 297.24 | -0.321 | 0.769 |
|  |  |  | ***Z*** | ***P-*value** |
| GWW (mm Hg%) | 40.00 (35.00) | 59.00 (43.00) | -0.586 | 0.558 |
| GWE (%) | 97.00 (2.00) | 96.00 (3.00) | -0.562 | 0.574 |

*GWI, global work index; GCW, global constructive work; GWW, global wasted work; GWE, global work efficiency.*

**SUPPLEMENTAL TABLE 2 │**Statistical comparisons of global myocardial work parameters between non-cardiovascular medication and cardiovascular medication groups in T2DM

| **Parameters** | **non-cardiovascular medication group**  **n=37** | **cardiovascular medication group**  **n=13** |  |  |
| --- | --- | --- | --- | --- |
|  |  |  | ***t*** | ***P-*value** |
| pulse pressure  (mm Hg) | 42.54 ± 9.25 | 38.31 ± 6.99 | -1.502 | 0.140 |

*PP, pulse pressure.*
